# Improving auditory attention decoding by classifying intracranial responses to glimpsed and masked acoustic events

**DOI:** 10.1162/imag_a_00148

**Published:** 2024-04-26

**Authors:** Vinay S. Raghavan, James O’Sullivan, Jose Herrero, Stephan Bickel, Ashesh D. Mehta, Nima Mesgarani

**Affiliations:** a Department of Electrical Engineering, Columbia University, New York, NY, United States; b Zuckerman Mind Brain Behavior Institute, Columbia University, New York, NY, United States; c The Feinstein Institutes for Medical Research, Northwell Health, Manhasset, NY, United States; d Department of Neurosurgery, Zucker School of Medicine at Hofstra/Northwell, Hempstead, NY, United States; e Department of Neurology, Zucker School of Medicine at Hofstra/Northwell, Hempstead, NY, United States

**Keywords:** intracranial EEG (iEEG), auditory attention decoding (AAD), event-related potential (ERP), glimpsing

## Abstract

Listeners with hearing loss have trouble following a conversation in multitalker environments. While modern hearing aids can generally amplify speech, these devices are unable to tune into a target speaker without first knowing to which speaker a user aims to attend. Brain-controlled hearing aids have been proposed using auditory attention decoding (AAD) methods, but current methods use the same model to compare the speech stimulus and neural response, regardless of the dynamic overlap between talkers which is known to influence neural encoding. Here, we propose a novel framework that directly classifies event-related potentials (ERPs) evoked by glimpsed and masked acoustic events to determine whether the source of the event was attended. We present a system that identifies auditory events using the local maxima in the envelope rate of change, assesses the temporal masking of auditory events relative to competing speakers, and utilizes masking-specific ERP classifiers to determine if the source of the event was attended. Using intracranial electrophysiological recordings, we showed that high gamma ERPs from recording sites in auditory cortex can effectively decode the attention of subjects. This method of AAD provides higher accuracy, shorter switch times, and more stable decoding results compared with traditional correlational methods, permitting the quick and accurate detection of changes in a listener’s attentional focus. This framework also holds unique potential for detecting instances of divided attention and inattention. Overall, we extend the scope of AAD algorithms by introducing the first linear, direct-classification method for determining a listener’s attentional focus that leverages the latest research in multitalker speech perception. This work represents another step toward informing the development of effective and intuitive brain-controlled hearing assistive devices.

## 1. INTRODUCTION

Individuals with hearing loss often find it difficult to attend to a single talker when multiple people speak simultaneously (Festen & Plomp, 1990; Peelle & Wingfield, 2016a, 2016b). While modern hearing aids can effectively filter out environmental noise, they cannot amplify the voice that the listener aims to attend to without knowing whose voice that is (Clark & Swanepoel, 2014; Green et al., 2022; Saki & Kehtarnavaz, 2016). Studies of multitalker speech perception have shown that neural signals contain an enhanced representation of the target speech stream, particularly in non-primary regions of the auditory cortex, including the superior temporal gyrus (STG) (Ding & Simon, 2012; Mesgarani & Chang, 2012; J. O’Sullivan et al., 2019; Zion Golumbic et al., 2013). This has given rise to the prospect of a “brain-controlled” hearing aid that uses auditory attention decoding (AAD) algorithms to determine the focus of a listener’s attention and provide selective enhancement of that speaker (Ceolini et al., 2020; Han et al., 2019; J. O’Sullivan et al., 2017; J. A. O’Sullivan et al., 2015).

Current methods for AAD that decode the attended speech stream are centered around linear models that aim to correlate stimulus features, such as the speech envelope, with the listener’s neural response (Alickovic et al., 2019; de Cheveigné et al., 2018; Wong et al., 2018). These methods are based on the knowledge that a listener’s neural response will be more correlated with the target talker due to an enhanced representation of this talker in the brain. Of these methods, canonical correlation analysis (CCA) has proven to be particularly effective and reproducible (Geirnaert, Vandecappelle, et al., 2021). However, these methods for AAD use the same algorithm to compute the stimulus-response correlation, regardless of the dynamic overlap between talkers which is known to influence neural encoding (Brodbeck et al., 2020; Raghavan et al., 2023).

While some adaptive AAD algorithms exist, they are fine-tuned on the timescale of minutes to enhance performance for a given subject or session, rather than on the timescale of milliseconds for a given decoding window (Geirnaert, Francart, et al, 2021; Geirnaert et al., 2022). Other methods have proposed separate decoding of segments at millisecond timescales; however, these segments were determined from the transient (Tanaka et al., 2023) and root-mean-square (RMS) intensity (Wang et al., 2021, 2022) levels of each talker separately. Recently, non-linear methods have been proposed which can, in theory, account for overlap between talkers and show increased decoding accuracy (Ciccarelli et al., 2019; Kuruvila et al., 2021; Lu et al., 2021; Xu et al., 2022). However, these methods are not interpretable and training them requires quantities of neural data that are often hard to acquire, sometimes leading to overfitting and a lack of architecture generalization to new datasets (Geirnaert, Vandecappelle, et al., 2021). Therefore, it may be helpful to apply linear methods for AAD to account for the overlap between talkers in a way that directly reflects neurally dissociable differences in speech processing.

Due to the natural fluctuations of speech, a multitalker scenario inherently contains glimpsed moments, that is, spectrotemporal regions in which one talker is louder than the others, and masked moments, that is, spectrotemporal regions in which one talker is quieter than the others (Cooke, 2006). Here, by “masked” moments, we specifically refer to energetic masking caused by overlap of stimuli at the auditory periphery, rather than informational masking caused by other means (Durlach et al., 2003; Kidd et al., 2016; Leek et al., 1991). Using fMRI, it has been shown that different types and degrees of masking activate different networks in the brain (Scott & McGettigan, 2013), and using iEEG, it was shown that masked phonemes in isolated words are restored at the acoustic-phonetic level in the auditory cortex (Leonard et al., 2016). A more recent iEEG study investigated whether continuous speech encoding in the auditory cortex depended on differences in relative overlap and revealed distinct encoding patterns for glimpsed and masked features of target speech in STG (Raghavan et al., 2023).

Separately, an iEEG study found that high-gamma band STG responses to single-talker speech are described more accurately by discrete events corresponding to the peaks in the rate of change of the speech envelope, termed peakRate events, compared to the speech envelope itself (Oganian & Chang, 2019). Initially, this suggests that discrete peakRate events, rather than the continuous speech envelope, may improve AAD performance. And together, these studies suggest that glimpsed and masked peakRate events could serve as robust features for improving attention decoding by focusing on the sections of the neural response most consistently influenced by the speech stimulus.

Furthermore, an AAD system must also be able to identify instances of other types of attention beyond selective attention (Shinn-Cunningham, 2008; Shinn-Cunningham & Best, 2008). In particular, it should be able to identify divided attention, in which attention is directed towards more than one auditory object (Agmon et al., 2022; Kaufman & Zion Golumbic, 2023; Lipschutz et al., 2002; Treisman & Davies, 2012) and inattention, when attention is not directed to any auditory objects in the environment (L. Cohen et al., 2021; Scheer et al., 2018). Current methods for AAD utilize a comparative approach by which the talker *most similar* to the listener’s neural response is deemed the target talker; however, this approach neglects instances in which attention is deployed differently. For example, a listener may want to monitor multiple sources simultaneously (Folds & Gerth, 1994). More commonly, various types of overlap occur naturally in conversational speech in which it is desirable to divide attention between multiple target talkers, including transitional, recognitional (Jefferson, 1984), and chordal (Schegloff, 2000) overlaps. One dataset of conversational German speech showed that 24% of segments were continuations from prior segments occurring during speech overlap (Meyer, 2023). Additionally, a listener may choose to direct their attention inward, away from any source in the environment, and thus, an AAD system should have the capacity to recognize this perceptual state as well. Therefore, an AAD system should be designed to detect each talker’s attentional status separately rather than assuming a single target and determining which talker is “more attended” than the others.

Given that non-invasive recording modalities have been struggling to overcome the inherent tradeoff between system accuracy and decoding delay (Geirnaert, Vandecappelle, et al., 2021; Geirnaert et al., 2020), we aimed to improve methods for AAD using iEEG, which has sufficient SNR to potentially enable a brain-controlled hearing aid capable of handling conversational speech. By leveraging distinct neural responses to acoustic events with differing overlap between speakers, this decoding framework aims to provide higher accuracy, shorter switch times, and less variable decoding results in a system capable of identifying different attention uses. To do so, we present a method for performing AAD based on classifying event-related potentials (ERPs) evoked by peakRate events, which we abbreviate as event-related potential classification (ERPC). We show that decoding attention through ERPC is enhanced when distinguishing between glimpsed and masked peakRate events. We then show that combining glimpsed and masked decoding strategies outperforms CCA, particularly at short decoding durations. Analyzing the dynamics of simulated switches of attention reveals that ERPC leads to faster and more stable detections of changes in attention. Finally, we show possible examples of how the classification approach taken by ERPC, as opposed to the typical comparison approach, holds unique potential for the detection of divided attention and inattention.

## 2. METHODS

### 2.1. Participants

Six subjects undergoing clinical treatment for epilepsy participated in this study. All subjects gave their written informed consent to participate in research before electrode implantation. All research protocols were approved and monitored by the institutional review boards at the Feinstein Institute for Medical Research, and all clinical investigation was conducted according to the principles expressed in the Declaration of Helsinki.

Measures were taken to minimize the potentially confounding effect of epilepsy on the data, including acquiring data from nonepileptic tissue, obtaining data several hours outside the window of seizures, and confirming no trials contained epileptic discharges (Parvizi & Kastner, 2018). These measures have been widely used in scientific explorations of the speech cortex in neurosurgical patients (Anumanchipalli et al., 2019; Bouchard et al., 2013; Edwards et al., 2010; Leuthardt et al., 2004; Mesgarani & Chang, 2012; Mesgarani et al., 2014; Tang et al., 2017). Two subjects were implanted with high-density subdural electrode arrays over the left temporal lobe with coverage of STG, and one of those subjects also had a depth electrode implanted in the left auditory cortex with coverage of HG. The remaining four subjects had depth electrodes implanted bilaterally, with varying amounts of coverage over the left and right auditory cortices for each subject (Fig. 2C). Of these six subjects, two were excluded for having recording sites that were not modulated by attention, as determined by the baseline CCA model performing worse than would be expected from non-invasive recordings (Geirnaert, Vandecappelle, et al., 2021). Notably, both of the excluded subjects did not have any coverage of STG.

### 2.2. Stimuli and experiments

Each participant took part in a two-talker experiment in which they were presented with one male talker and one female talker matched to have the same root-mean-square intensity and with no spatial separation between them. Both the male and female talkers were native American-English speakers with an average F0 of 65 Hz and 175 Hz, respectively. The speech material consisted of stories about various topics and was recorded in-house. All stimuli were presented using a single Bose SoundLink Mini 2 speaker situated directly in front of the subject. The sound level was adjusted for each subject to be at a comfortable level between 60–70 dB SPL.

The experiment was divided into four blocks. Before each block, the subject was instructed to focus their attention on one talker and ignore the other. All subjects began the experiment by attending to the male talker and switched their attention to the alternate talker on each subsequent block. Each block was semantically continuous, such that a full story was heard before switching attention to the alternate talker, to provide a naturalistic listening scenario and aid the participant’s ability to focus on the target. The stories were intermittently paused at sentence breaks in the target talker after the target had spoken between three and five sentence. The locations of the pauses were predetermined and the same for all subjects, but the subjects were unaware of when the pauses would occur. The stories were paused on average every 20 s (min 9 s, max 30 s) for a total of 11 m 37 s of audio divided into 35 trials. Subjects were asked to repeat the last sentence of the target talker to ensure engagement with the task. All subjects performed well on this task (mean = 90%, standard deviation = 8%, minimum = 80%), so no data were excluded due to poor performance.

### 2.3. Neural data hardware and preprocessing

Most subjects at NSUH were recorded using Tucker Davis Technologies (TDT; Alachua, FL) hardware and sampled at 2441 Hz. One subject was recorded using Xltek (Natus, San Carlos, CA) hardware and sampled at 500 Hz, and another subject was recorded using Blackrock Neurotech (Salt Lake City, UT) hardware and sampled at 3 kHz. All further processing steps were performed offline. All filters were designed using MATLAB’s Filter Design Toolbox and were used in both forward and backward directions to remove phase distortion. The TDT and Blackrock data were resampled to 500 Hz. A 1st-order Butterworth high-pass filter with a cut-off frequency at 1 Hz was used to remove DC drift. Data were subsequently re-referenced using a local scheme whereby each electrode was referenced relative to its nearest neighbors. Line noise at 60 Hz and its harmonics (up to 240 Hz) were removed using 2nd-order IIR notch filters with a bandwidth of 1 Hz. A period of silence lasting 1 min was recorded before the multitalker experiment, and the data were normalized by subtracting the mean and dividing by the standard deviation of this pre-stimulus period.

The data were then filtered into the high-gamma band (70–150 Hz), which is correlated with multi-unit firing rates (Ray & Maunsell, 2011), the envelope of which is known to be modulated by speech. To obtain the envelope of this broadband signal, we first filtered the data into 8 frequency bands between 70 and 150 Hz, each with a bandwidth of 10 Hz, using Chebyshev Type 2 filters. Then, the envelope of each band was obtained by taking the magnitude of the analytic signal obtained via the Hilbert transform. We took the average of all eight frequency bands as the final envelope and resampled this response to 100 Hz. This multi-band approach is commonly used for the extraction of the high-gamma envelope (Bouchard et al., 2013; Edwards et al., 2010), as the magnitude of the analytic signal should only be used to extract the envelope from a narrowband signal such that spectra of the envelope and phase signals are non-overlapping (Ktonas & Papp, 1980). Electrodes were tested for speech responsiveness by calculating the effect size (Cohen’s D) between the distributions of the responses during speech and silence (J. Cohen, 2013). Non-epileptic electrodes with an effect size greater than 0.2—considered a small but significant effect size—were retained for further analysis. In total, 174 speech-responsive electrodes in and around auditory cortex were analyzed, including those in superior temporal gyrus (59), Heschl’s gyrus (52), middle temporal gyrus (27), transverse temporal sulcus (20), planum temporale (8), insula (6), and superior temporal sulcus (2).

#### 2.3.1. Plotting electrodes on an average brain

The electrodes were first mapped onto the brain of each subject using co-registration by iELVis (Groppe et al., 2017), followed by their identification on the post-implantation CT scan using BioImage Suite (Papademetris et al., 2006). To obtain the anatomical location labels of these electrodes, we used Freesurfer’s automated cortical parcellation (Dykstra et al., 2012; Fischl, 2004; Fischl et al., 1999) by the Destrieux brain atlas (Destrieux et al., 2010). These labels were closely inspected by the neurosurgeons using the subject’s co-registered post-implant MRI. Electrodes were plotted on the average Freesurfer brain template.

### 2.4. Audio data feature extraction

Auditory spectrograms were computed from the raw waveforms of each talker sampled at 16 kHz using a python implementation of the *wav2aud* function of the NSL toolbox (Mischler et al., 2023; Yang et al., 1992). A frame length of 8 ms and leaky integration time constant of 4 ms are used, resulting in spectrograms with time bins every 10 ms, and 100 constant Q-filters logarithmically spaced between 50 Hz and 8 kHz are used, resulting in 100 frequency bins. The speech envelopes used for all CCA analyses were then computed by taking the sum of each frequency bin of the spectrogram at each point in time, resulting in an envelope with a sampling frequency of 100 Hz. Any periods of silence at the beginning and end of the experiments were removed.

To extract peakRate events, we first bandpass filtered the speech envelope between 1–10 Hz using a 3rd-order Butterworth filter applied in both the forward and backward direction to prevent phase shift. Then, we calculated the positive rate-of-change of the envelope as the half-wave rectified derivative of the filtered envelope. Finally, peakRate events were defined as the time series of local peaks of the positive rate-of-change of the envelope, as in previous work (Oganian & Chang, 2019). Unlike previous work, we chose to apply a threshold to the peakRate events, as we only wanted to consider prominent events, and our model did not incorporate the rate magnitude of different events. We used a threshold equal to 0.1 σ, where σ is the standard deviation of the positive rate-of-change of the envelope, to be robust to differences in scale. This threshold removed the smallest 6% of peakRate events.

#### 2.4.1. Glimpsed and masked peakRate events

We assessed the spectrotemporal masking around each peakRate event to allocate it as either a glimpsed or masked event. To do so, we first defined a window of time [−200, 200] ms around each peakRate event as the window over which the glimpse and mask ratios would be computed. This window was based on previous work showing the average spectrogram surrounding peakRate events primarily deviates from its average magnitude in this [−200, 200] ms range (Oganian & Chang, 2019). The glimpse ratio for a peakRate event for a given talker is defined as the percentage of time-frequency bins of the talker’s spectrogram that have a magnitude greater than or equal to the magnitude of the corresponding time-frequency bins of the background spectrogram by the glimpse SNR (Brungart et al., 2006; Cooke, 2006). For a talker *T* and all other sources in the background *B*, this can be given by $\text{rati}o_{G}=\frac{\left|S_{T}\geq S_{B}*SNR_{G}\right|}{\left|S_{T}\right|}$, where *ratio*_*G*_ is the glimpse ratio, *S*_*T*_ is the talker spectrogram, *S*_*B*_ is the background spectrogram, and *SNR*_*G*_ is the glimpse SNR. Similarly, the mask ratio is defined as the percentage of time-frequency bins of the talker’s spectrogram that have magnitude less than the corresponding time-frequency bins of the background spectrogram by the glimpse SNR, such that the glimpse and mask ratios of each event sum to 1. We use a glimpse SNR of −4 dB throughout, in line with previous work showing that this threshold separates glimpsed and masked representations in a way that maximizes their predictive power (Raghavan et al., 2023). By this definition, each time-frequency bin of a talker’s spectrogram could have a smaller magnitude than the corresponding time-frequency bin of the background by up to 4 dB and still be considered glimpsed. This procedure results in an average glimpse ratio of 0.63 ± 0.21 (mean ± s.d.) and an average mask ratio of 0.37 ± 0.20 over all peakRate events.

### 2.5. Canonical correlation analysis decoding system

**Fig. 1.**
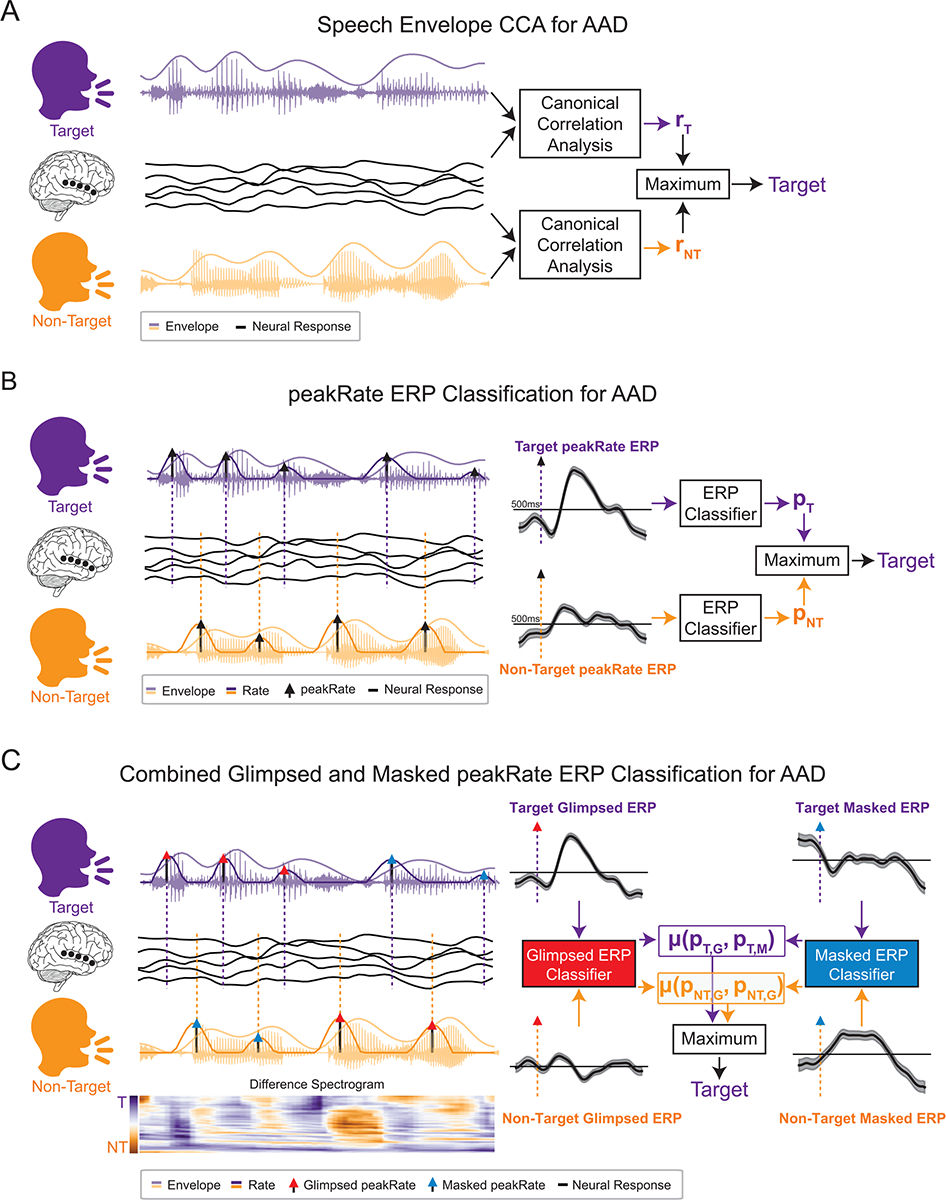
Schematics of auditory attention decoding (AAD) methods. (A) Schematic of AAD using canonical correlation analysis (CCA). The speech envelopes of both talkers are compared with the listener’s neural response (r_T_: target canonical correlation; r_NT_: non-target canonical correlation), and the talker with the higher canonical correlation is determined to be the target. (B) Schematic of AAD using event-related potential classification (ERPC). peakRate events are extracted from the speech envelope of both talkers and used to compute average ERPs aligned to each event. Vertical dashed lines indicate peakRate event times for target (purple) and non-target (yellow) talkers. The ERPs are classified (p_T_: probability that target talker is attended; p_NT_: probability that non-target talker is attended), and the talker with a higher probability of being attended is determined to be the target. (C) Schematic of AAD using glimpsed and masked ERPC. peakRate events from each talker are allocated as glimpsed (red arrowhead) or masked (blue arrowhead) events depending on the relative loudness of the talkers (difference spectrogram). Each of the average glimpsed ERPs and masked ERPs are separately classified (p_T,G_: probability glimpsed target events are attended; p_T,M_: probability masked target events are attended; p_NT,G_: probability glimpsed non-target events are attended; p_NT,M_: probability masked non-target events are attended), and the talker with the higher average probability of being attended is determined to be the target. Example target and non-target ERPs shown here are the mean and standard error over all electrodes in STG.

The baseline model used as the current standard for determining the target talker in an AAD paradigm utilizes canonical correlation analysis (CCA) (Fig. 1A) (de Cheveigné et al., 2018). This method, as used here, learns to apply a spatial filter to the neural recordings and a temporal filter to the speech envelope to project both the neural and acoustic representations into a new domain in which they are maximally correlated. CCA has been shown to outperform other linear, stimulus-reconstruction approaches to AAD using EEG (Geirnaert, Vandecappelle, et al., 2021). Here, we utilized time-lags of [−500, 0] ms of the speech envelope, no additional time lags for the neural responses, and evaluated the correlation between these data using the top canonical component. This resulted in the neural input dimensionality equal to *N*_*ch*_, the envelope input dimensionality equal to (*t*_*min*_ *− t*_*max*_)* *f*_*s*_ + 1, and the canonical component dimensionality equal to 1, where *N*_*ch*_ is the number of neural recording channels, *f*_*s*_ = 100 is the sampling frequency, and *t*_*min*_ and *t*_*max*_ are the minimum and maximum time lags, respectively. The results were evaluated separately for each subject using 5-fold cross-validation, where each fold consisted of exactly 7 trials with a fold duration between 149.9–150.3 s.

To evaluate the performance of this system, we produce the performance curve which shows the accuracy of the attention decoding system as a function of the window duration of data used to calculate the decision. First, we applied the learned CCA model to the entire test data to compute the canonical component of the test data over time. We then took segments of the test data component of a given duration and compared the correlation of the target talker’s canonical components with the correlation of the non-target talker’s canonical components. If the correlation of the target talker’s canonical components exceeded the correlation of the non-target talker’s canonical components, then that segment was determined to be decoded correctly. Note that the test data segments over which the correlation was computed were overlapping, such that the accuracy directly represents the percentage of time steps correctly classified in a continuously operating system. This allows for a direct comparison between CCA and ERPC decoding.

### 2.6. Event-related potential classification (ERPC) decoding system

Our proposed model for determining the target talker in an AAD paradigm is based on using a classifier to predict whether an ERP to a peakRate event was evoked by a target or non-target talker (Fig. 1B). First, this method computes the average ERP for each electrode over all events contained within a segment of a given duration. Then, it classifies these average ERPs and produces a probability that they were evoked by a target talker using Platt scaling (Platt, 1999). The general peakRate classifier uses all extracted peakRate events, the final glimpsed peakRate classifier uses all extracted peakRate events with a glimpse ratio greater than 0.9, and the final masked peakRate classifier uses all extracted peakRate events with a mask ratio greater than 0.8. Note that events are not included within a window until their glimpse and mask ratios can be computed that is, after 200 ms. We utilize ERPs of [0, 500] ms around the peakRate events and classify these responses using a support vector machine classifier using a radial basis function kernel with a regularization value of C = 10 implemented in scikit-learn (Pedregosa et al., 2011). Thus, the dimensionality of the input is equal to *N*_*ch*_ * ((*t*_*max*_ − *t*_*min*_) * *f*_*s*_ + 1). The results were evaluated separately for each subject using the same 5-fold cross-validation as with CCA.

To evaluate the performance curve, average ERPs are computed and classified over varying window durations. All models were trained with average ERPs aggregated using the same window length as used during testing, meaning that each duration utilized a separately trained model. A given set of ERPs was only included in the train/test set once, even if that same set of ERPs was present for multiple windows. During the test phase, we computed the accuracy at a given duration based on the percentage of target talker windows correctly determined to be the target and the percentage of non-target talker windows correctly determined to be the non-target. Therefore, the accuracy here represents the average classifier test accuracy rather than the AAD accuracy in a continuously operating system.

#### 2.6.1. Joint classification of glimpsed and masked ERPs

To combine the glimpsed and masked ERP classifiers, we take the average of both classifiers to produce a final prediction used to decode the listener’s attention (Fig. 1C). For a decoding window of a given duration, we first extract and evaluate all of the glimpsed and masked peakRate ERPs using the respective classifiers for both target and non-target talkers. We then take the average between the glimpsed and masked classifiers for both talkers to produce the overall probability for each talker. If a window contained only one type of event, then that classifier probability was used as the overall probability. Next, we compared the ERPC probability between talkers, such that the window was decoded correctly if the target talker had a higher probability of being attended than the non-target. If a window contained events from only one talker, then the probability from that talker alone was used, that is, if the target or non-target had a greater or less than 50% probability of being attended, respectively, the window was decoded correctly.

If a window did not contain any peakRate events crossing the glimpse or mask thresholds, then the window was assigned the same probability as the previous window, that is, decoding decisions are held until the next decision can be made. This was done to produce an accuracy most representative of how the decoder would operate in practice, such that the accuracy directly represents the percentage of time steps correctly classified in a continuously operating system. This also allows a direct comparison with CCA decoding.

We also sought to evaluate the performance of an ERPC system capable of producing new decisions at each point in time. Therefore, we also tested a system in which windows were evaluated using the glimpsed and masked classifiers when possible but used CCA as a fallback when no events were sufficiently glimpsed/masked, instead of holding the previous decision. This allows us to test whether improvements seen with ERPC were due to a more effective decoding strategy, an ability to focus on the most stereotyped segments of the neural response, or both.

### 2.7. Dynamic switching of attention

To assess a simulated dynamic switching of attention, we concatenated the data for each trial within each test fold but switched the identity of the target and non-target talker for every other trial. This made it such that the simulated attention switch moments corresponded to trial boundaries, and moments of simulated sustained attention corresponded to sustained attention during the experiment. We chose trial boundaries as the attention switch point because attention switching often happens at salient moments alongside onsets (Huang & Elhilali, 2017, 2020). While onsets show unique responses in LFPs (Chalas et al., 2023), high gamma onset responses in the auditory cortex occur for any pause longer than 200 ms, a common occurrence between phrases or sentences in continuous speech (Hamilton et al., 2018).

This simulation makes it possible to visualize how different methods for AAD may behave in situations with an attention switch. The CCA results show the correlation between each speaker’s canonical components over time. The ERPC results show the predicted probability that each speaker is the target over time. While the CCA results are continuous, as correlation can be computed over a window at each point in time, the ERPC results are discrete. In particular, a new ERPC result is only produced when sufficiently glimpsed or masked events are present. Therefore, the time points in which no new ERPC decision is made are held from the previous decision.

#### 2.7.1. Average switch time course, latency, and stability

To compute the average time course around the attention switch, the CCA correlations and ERPC probabilities were averaged over all simulated switches for each decoding window duration for each subject. This resulted in 30 simulated switches per window per subject, as each fold of the 5-fold cross-validation contained 7 trials and, therefore, 6 switches. To compute the average switch time, the average time course around the attention switch was produced for each subject. We then measured the amount of time it took after the simulated switch for the model to identify the opposite talker as more likely to be the target. This was done for each decoding window duration and for each subject, then averaged over subjects. To determine the stability of the decoded time course, we measured the distribution of the amount of time between decoded switches of attention for each method and compared this with the distribution of the amount of time between simulated switches of attention.

## 3. RESULTS

### 3.1. Envelope CCA outperforms generic peakRate ERPC

We first computed the baseline performance for each subject using the CCA method with the speech envelope and the ERPC method with peakRate events to determine how these methods compare in their ability to determine the target talker. For CCA, this was done by computing the first pair of canonical components, segmenting them into windows of various durations, and determining whether the Pearson’s correlation between components was higher for the target or non-target talker. For ERPC, this was done by segmenting the target and non-target stimuli into windows of various durations, computing the average ERP over that window at each electrode, and classifying whether those ERPs were evoked by the target or non-target talker.

**Fig. 2.**
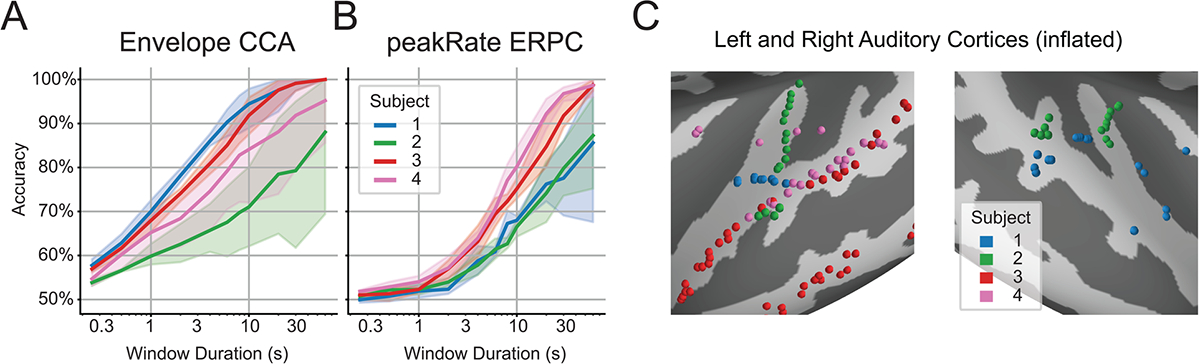
Baseline AAD results and electrode locations. (A) Baseline AAD performance with CCA using the speech envelope. (B) Baseline AAD performance with ERPC using all peakRate events. (C) Electrode locations of all subjects over the left and right auditory cortices.

For all subjects at nearly all window durations, CCA outperforms ERPC in decoding accuracy (Fig. 2A–B). This demonstrates how effective CCA is for correlating the speech envelope with neural responses. We also observe variation in subject performance, particularly in that CCA performs well in subjects with broad coverage while ERPC performs well in subjects with STG coverage (Fig. 2C). Successive results will present the average and standard error over these subjects.

### 3.2. Glimpsed and masked peakRate ERPC outperforms CCA

**Fig. 3.**
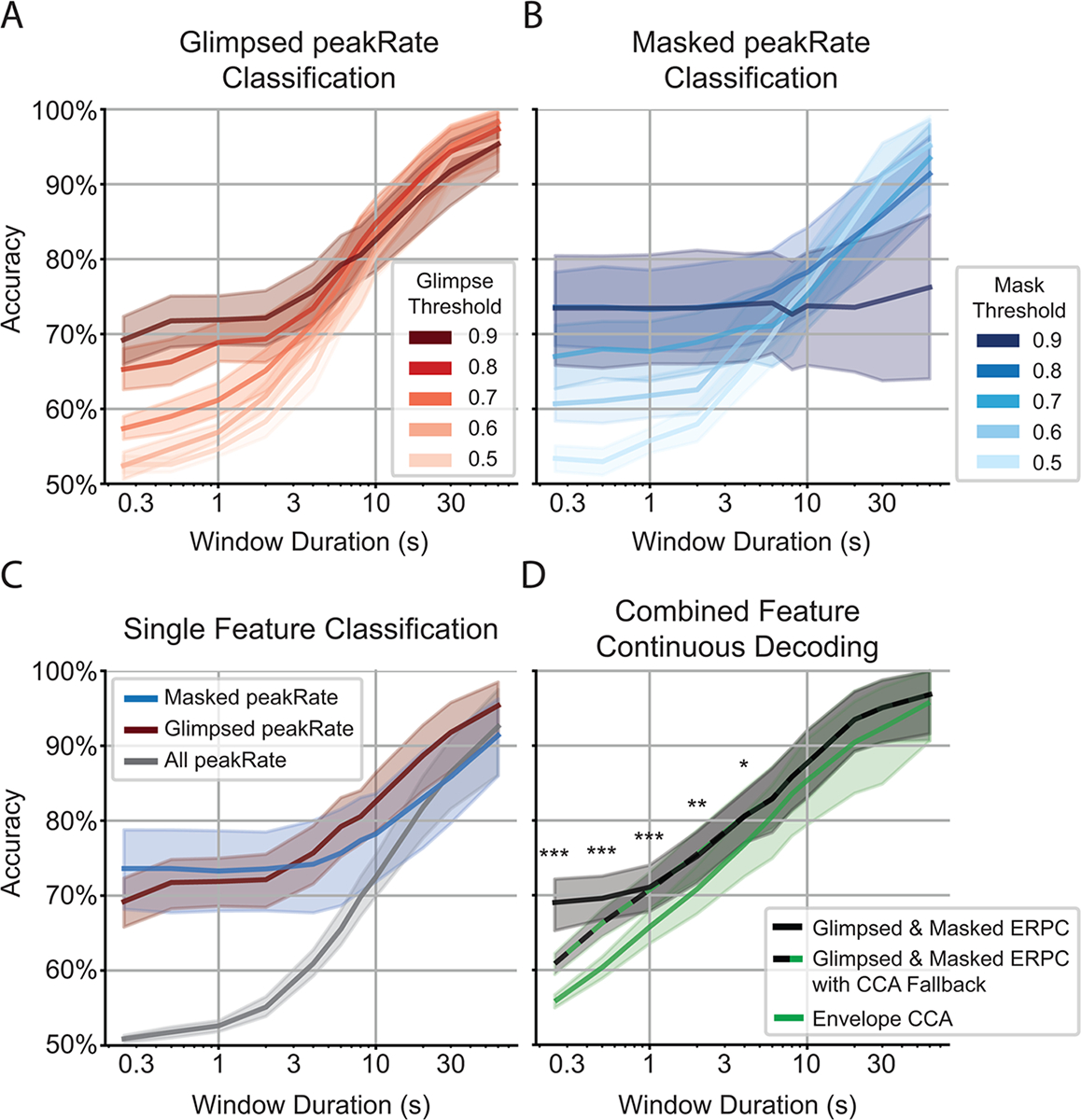
Comparison of performance curves for different AAD configurations. (A) Classification accuracies for ERPC using glimpsed peakRate events over different glimpse threshold values. (B) Classification accuracies for ERPC using masked peakRate events over different mask threshold values. (C) Comparison of all single-feature classification accuracies. Attention decoding is poor when using all peakRate events but improves when distinguishing between glimpsed and masked peakRate events, especially at short latencies. Glimpsed peakRate classification uses a glimpse threshold of 0.9, and masked peakRate classification uses a mask threshold of 0.8. (D) Comparison of aggregate decoding systems. Combining the glimpsed and masked classifiers significantly outperforms CCA across window sizes at and below 4 s (*: p < 0.05, **: p < 0.01, ***: p < 0.001; Bonferroni-corrected paired t-test). Because ERPC maintains the prior decoding decision at time steps lacking sufficiently glimpsed/masked events, CCA can be used as a fallback. Using ERPC to classify and hold the decisions from windows with sufficient events results in the highest overall accuracy.

Because we expect differences in the neural encoding of multitalker speech depending on the degree of masking, we distinguished between glimpsed and masked peakRate events, then separately classified the ERPs to each of those events. To determine the degree to which an event must be glimpsed or masked, we performed ERPC on events of varying glimpse and mask ratio thresholds (Fig. 3A–B). This revealed that the highest classification accuracy at low latencies could be achieved for the most glimpsed peakRate events with a glimpse ratio threshold of 0.9, indicating >90% of the time-frequency bins of the talker’s spectrogram had a sufficiently greater magnitude than the background (see Section 2.4.1). On the other hand, masked peakRate events only required a mask ratio threshold of 0.8 to be classified with high accuracy at low latencies. Comparing our results so far (Fig. 3C), we see that partitioning all peakRates into glimpsed and masked events and setting sufficient thresholds on the glimpse and mask ratios allows us to considerably outperform ERPC using all peakRate events.

Given that ERPC using glimpsed and masked peakRate events separately performs well, we then combined these decoding strategies, as they utilize independent sets of events and, thus, should be complementary rather than redundant. Indeed, we observe that by averaging the classification probabilities between the glimpsed and masked classifiers at each decoding window, this method of decoding outperforms CCA across all decoding window durations, especially in the shortest windows that represent a crucial operating regime of an AAD system (Fig. 3D).

While it appears to be advantageous to only make a decoding decision in the windows in which there are events that are sufficiently glimpsed or masked, it may also be desirable to operate a system that can make new decoding decisions at every point in time, regardless of the degree of masking in the decoding window, rather than holding previous decisions. For that reason, we also evaluate the results of a model that performs combined glimpsed and masked ERPC while also using CCA as a fallback for windows lacking sufficiently glimpsed/masked events (Fig. 3D). This method still outperforms CCA at all decoding window durations, and it suggests that the performance improvement that ERPC achieves for short decoding windows can be attributed both to a better decoding strategy for glimpsed and masked windows and ignoring the windows that do not show the most stereotypical responses.

### 3.3. ERPC can decode simulated attention switches faster and with more stability

**Fig. 4.**
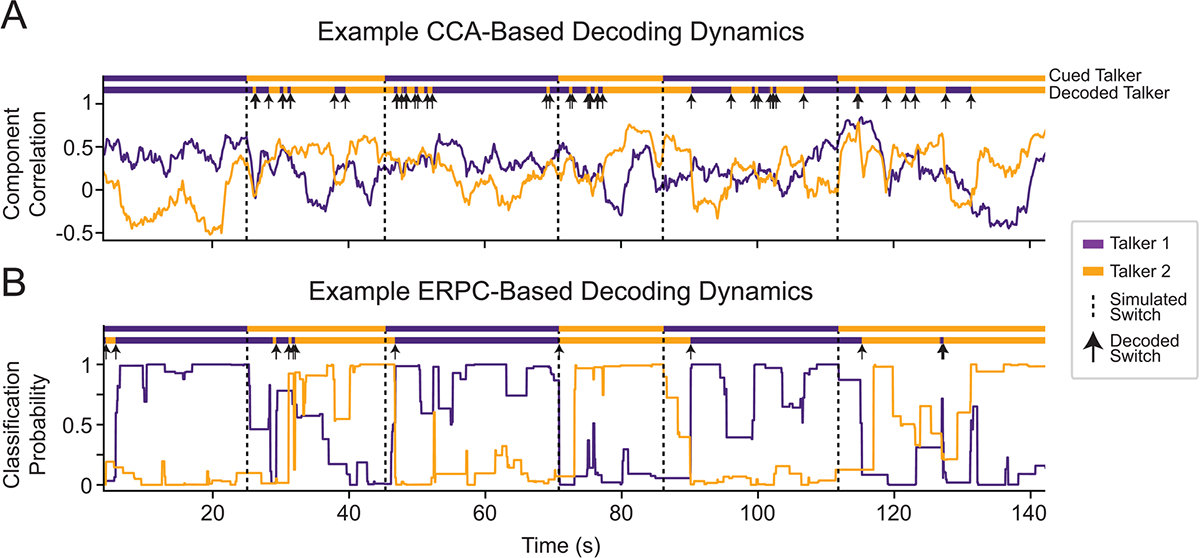
A simulated example of dynamic attention switching in subject 3 using a 4-s decoding window. (A) CCA decoding dynamics show the correlation between the canonical components produced by CCA over time. Instances in which correlation values are similar lead to an undesirable rapid switching between talkers. (B) ERPC decoding dynamics show the predicted probability that the average ERP was evoked by a target talker over time. By relying only on the most discriminable sections of the neural responses, ERPC dynamics are more accurate and stable.

With the increased decoding accuracy achieved by combining glimpsed and masked ERPC, we sought to understand its decoding dynamics. In particular, we wanted to characterize how this system would respond to simulated switches of attention, as previously investigated (J. O’Sullivan et al., 2017), as any brain-controlled hearing aid must be able to tracking the shifting attention of a listener. Figure 4 contains an example of the decoding dynamics of a CCA system (top) and ERPC system (bottom) for the same period containing simulated switches of attention. In the CCA decoding dynamics, the periods around the attention switches contain many incorrect decoding decisions and show slow reactions to the attention switches, for example, around t = 45 s in Figure 4. Meanwhile, in the ERPC decoding dynamics, the decoded switch occurs in a relatively quick and robust way. In the CCA decoding dynamics, the component correlations partially depend on the correlation between talkers, leading to highly variable decoding decisions when correlations are low, for example, around t = 75 s in Figure 4. Meanwhile, in the ERPC decoding dynamics, the classification probabilities are only generated for highly stereotyped events, leading to less variable decoding decisions. The decoding dynamics shown here are similar to those seen in other participants and were chosen to exemplify differences between ERPC and CCA.

**Fig. 5.**
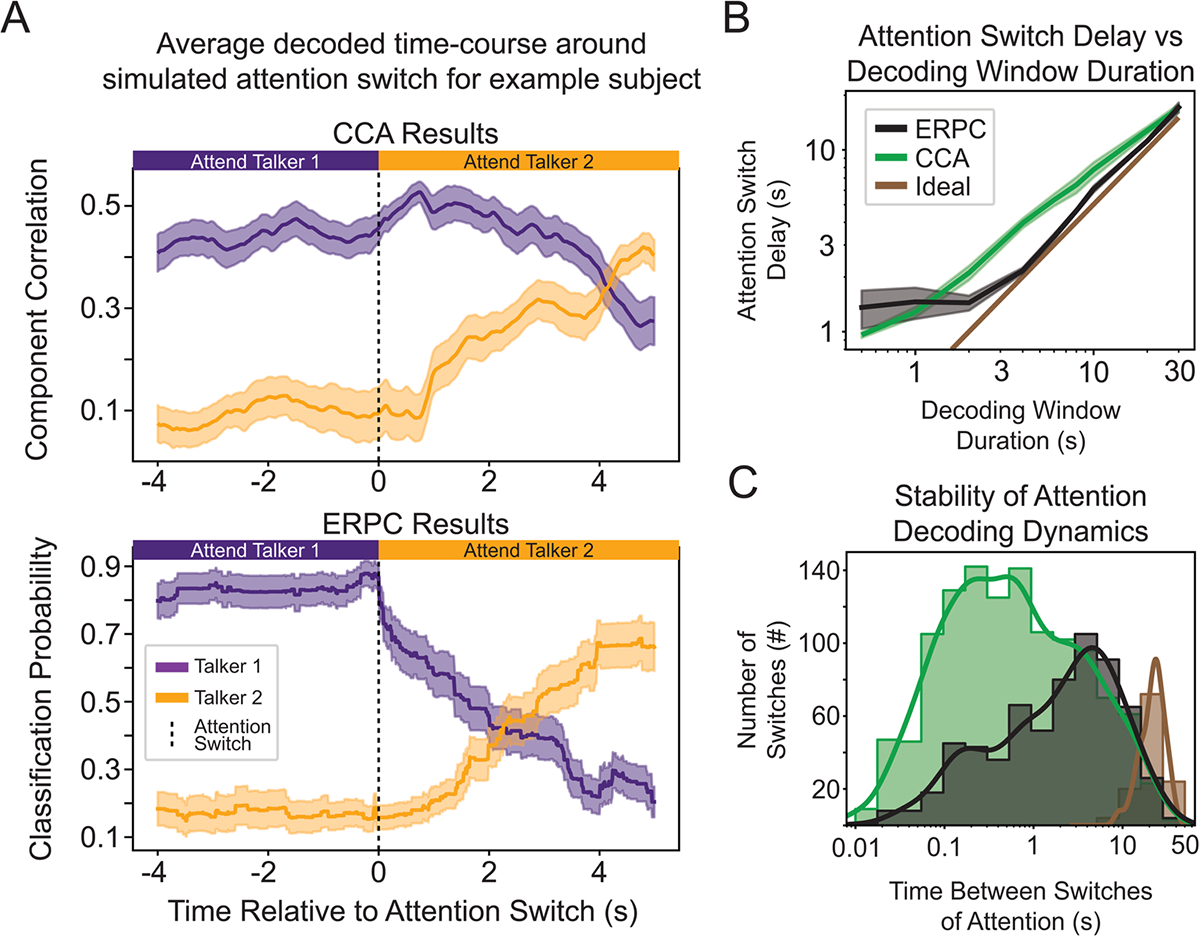
Properties of attention-switching dynamics. (A) Time-course of decoding around the switch of attention for Subject 3 using a 4-s decoding window averaged over simulated attention switch events. CCA takes around 4 s to detect the switch (top), while ERPC takes around 2 s (bottom). (B) Time needed to detect a switch of attention over different decoding window lengths averaged over all subjects. ERPC detects switches more rapidly than CCA for window sizes greater than 1 s. “Ideal” corresponds to a delay equal to half of the decoding window duration. (C) Comparison of decoding stability with simulated switching data for all subjects. CCA contains many more decoded switches in attention with short durations between switches, indicating relatively unstable decoding decisions. “Ideal” corresponds to the distribution of simulated switches.

In order to better evaluate these systems, we quantified the switch time and stability of the CCA and ERPC systems. First, switch time is an important property of any AAD model, as the system needs to react rapidly to changes in the user’s focus of attention. Therefore, to quantify the switch time, we computed the average decoded time course around the switch and found the point in time after the switch at which the new target talker was decoded as the target. Figure 5A contains the average decoded time course around the switch for subject 3 using a 4 s decoding window, showing how ERPC takes around 2 s to detect the switch (bottom), while CCA takes around 4 s (top). These estimated switch times were computed for each decoding window duration (Fig. 5B) and reveal that CCA detects attention switches slower than ERPC for decoding window durations greater than 1 s. At 1 s and below, ERPC switch times are slower and highly variable. This is likely due to ERPC attention switches being dependent on the presence of highly glimpsed or masked events immediately after the switch, which is random in this dataset.

Next, AAD methods may incorrectly detect a switch in attention; therefore, it is essential to know how often these incorrect switches occur. If the time between these switches is very brief, it could result in rapid changes in AAD amplification that may be undesirable for a listener. Therefore, to compare the decoding stability of these methods, we measured the distribution of the time between decoded attention switches, regardless of whether they were correct. This was done by noting all the decoded switch times, computing the duration between adjacent switches, and modeling the distribution of these durations. This allowed us to produce a histogram showing how long a given talker was decoded as the target before the system switched to the opposite talker (Fig. 5C). When using a 4 s decoding window, neither CCA nor ERPC is close to the true distribution of attention switch durations; nevertheless, CCA contains considerably more attention switches under 4 s, indicating the instability of this method.

### 3.4. ERPC holds the capacity to handle cases of divided attention and inattention

**Fig. 6.**
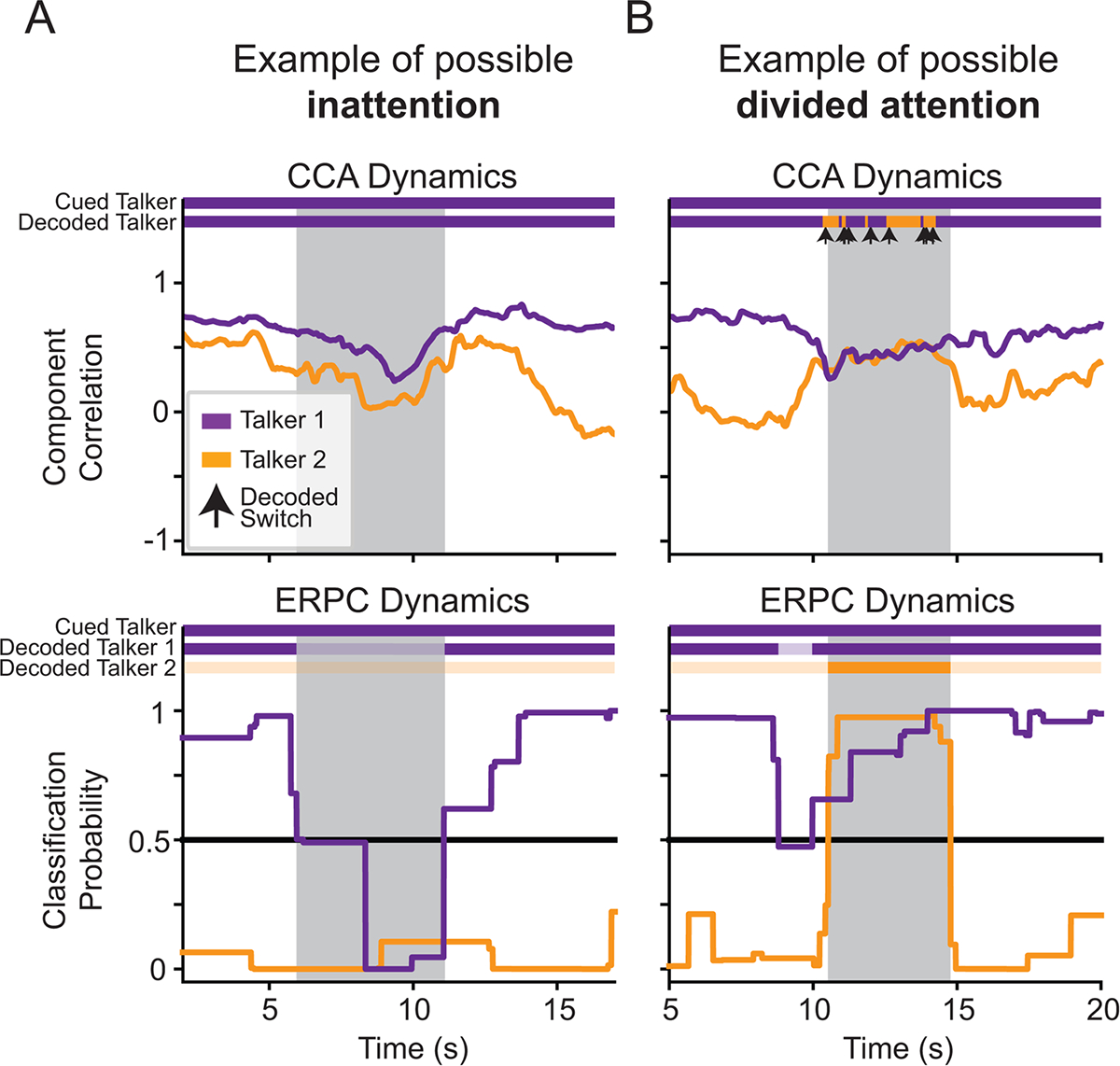
Possible examples of atypical uses of attention in the decoded dynamics of Subject 1. (A) An example of a possible moment of inattention (shaded area). In the CCA dynamics (top), the correlations with both talkers drop simultaneously, but due to the comparative nature of CCA decoding, the talker with the higher correlation is decoded as the target. In the ERPC dynamics (bottom), both talkers are determined to have a <50% probability of being the target, and thus, an AAD system might know to attenuate all external sources. (B) An example of a possible moment of divided attention (shaded area). In the CCA dynamics (top), the correlations with both talkers appear similar, but due to the comparative nature of CCA decoding, the system oscillates between talkers. In the ERPC dynamics (bottom), both talkers are determined to have a >50% probability of being the target, and thus, an AAD system might know to enhance both sources.

Unlike CCA and other stimulus-response correlation methods, which produce a Pearson’s correlation “score” for each talker, ERPC produces a probability that the classified ERPs were evoked by the target talker. This is useful because it holds the capacity to determine if a listener is distributing their attention between talkers or is not attending to any talkers, in addition to determining if a listener is selectively attending to one talker. Figure 6 shows possible examples of what these atypical uses of attention might look like in the decoding dynamics of CCA and ERPC.

In Figure 6A, we show a possible period of inattention around 6–11 s. In the CCA dynamics, the correlation of both talkers drops; however, the purple talker still has the higher correlation and, thus, is decoded as the target. In the ERPC dynamics, both the ERPs evoked by both talkers were consistent with ERPs to non-target speech, and thus, both talkers are decoded as non-targets.

In Figure 6B, we show a possible period of divided attention around 10–15 s. In the CCA dynamics, the correlations of both talkers are relatively high and similar; therefore, due to the comparative nature of CCA decoding, the system alternates in its decoding decision between talkers. In the ERPC dynamics, the ERPs evoked by both talkers were consistent with ERPs to target speech, and thus, both talkers are decoded as targets.

## 4. DISCUSSION

We have presented a novel method for performing auditory attention decoding (AAD) based on the classification of ERPs evoked by peakRate events. We showed that decoding attention through ERPC is enhanced by distinguishing between glimpsed and masked peakRate events. We then showed that combining glimpsed and masked decoding strategies outperform CCA, particularly at short decoding durations. Next, we analyzed the dynamics of simulated switches of attention, showing that ERPC leads to generally faster detections of changes in attention and more stable decoding decisions, compared to CCA. Finally, we gave possible examples showing ERPC holds the capacity for the detection of divided attention and inattention. These results improve upon methods for AAD to develop a system capable of meeting the demands of conversational speech and handling atypical uses of attention.

### 4.1. Direct-classification approach to AAD enables the detection of different types of attention

Even though direct-classification approaches are more common among brain-computer interfaces (BCIs) that make discrete decisions (Lotte et al., 2018), direct-classification approaches in AAD are relatively uncommon. Until now, no linear and only one non-linear direct-classification approaches to attended speaker identity decoding have been reported. This approach trained a convolutional neural network (CNN) to produce a “similarity score” between a talker’s speech envelope and the listener’s neural response (Ciccarelli et al., 2019). While this similarity score could have been interpreted as a probability, this work instead took a comparative approach to draw a direct comparison with stimulus reconstruction.

By utilizing direct classification, the aforementioned non-linear approach and the linear approach presented here offer unique benefits to solving the AAD problem. In particular, direct classification allows us to assign each talker a probability of being a target, irrespective of the decoded status of other talkers. This allows the system to detect instances of inattention, during which the listener is ignoring all talkers or has an inward focus of attention, preventing an AAD system from incorrectly amplifying a talker to whom the listener does not aim to attend. This also allows the system to detect instances of divided attention, during which the listener is attending to multiple talkers that may contain overlap. Given that natural conversations often contain various forms of overlap (Jefferson, 1984; Schegloff, 2000), which occur to different degrees in different cultures (Tannen, 2012), it is essential that a brain-controlled hearing aid be able to provide appropriate amplification in these scenarios.

### 4.2. Decoding talker identity rather than location

Despite the paucity of direct classification approaches to talker identity decoding, many non-linear direct classification approaches have been reported that aim to decode the locus of attention (LoA) (Pahuja et al., 2023; Su et al., 2022; Vandecappelle et al., 2021; Zhang et al., 2023). While decoding the LoA can often be done quite accurately, this method often requires large spatial differences to work well and breaks down when a non-target sound source is co-directional with the target. In fact, recent research has suggested that even linear LoA decoding methods are decoding the eye gaze and trial fingerprints, rather than neural signatures of spatial attention (Rotaru et al., 2024). Furthermore, listeners with hearing loss have been shown to have poorer spatial acuity and cortical responses less modulated by spatial attention compared to normal-hearing counterparts, suggesting that LoA decoding may not be as effective on the listeners who need it most (Dai et al., 2018). On the other hand, it has been shown that neural envelope tracking of target speech in multitalker settings is selectively enhanced in listeners with hearing loss (Decruy et al., 2020; Fuglsang et al., 2020). Therefore, it is most important to develop methods for AAD that decode the talker identity, rather than the talker location, as these methods will be most applicable to listeners with hearing loss and do not suffer from gaze-related confounds.

### 4.3. A new conceptualization of attention switches minimizes this bottleneck

Because direct classification allows the development of an AAD system that is permitted to amplify multiple talkers simultaneously, this changes the way the problem of attention switching is treated by an AAD system. Comparative AAD methods, such as CCA, can only amplify one talker at a time; therefore, these methods must track every turn in a conversation, forced to detect the rapid back-and-forths of a lively conversation. This proves to be a challenging task, as average utterance durations during turns in conversational speech last between 2–6 s (Chapple, 1939; Meyer, 2023). On the other hand, direct classification treats each talker independently and, thus, can consider all participants in a conversation as targets. Therefore, “attention switches” only need to occur when a talker is joining or leaving a conversation or when the listener changes focus to an entirely new conversation. This framework is advantageous because these instances of attention-switching between conversations occur considerably less often than turns within a conversation, thus limiting the influence of one of the biggest bottlenecks in the implementation of a smart hearing aid utilizing AAD.

In addition to conversation-driven changes in attention, it may also be important to rapidly enhance sounds that attract a listener’s attention in a bottom-up manner. Salient auditory events that attract attention are primarily driven by loudness in addition to other acoustic characteristics (Huang & Elhilali, 2017). It follows that these events would be highly glimpsed and, thus, suitable for classification. While our results indicate that ERPC may take longer than CCA to detect an attention switch with decoding windows <1 s, these results were produced using artificial attention switches containing events with random amounts of masking at the attention switch. In realistic scenarios, attention switches are likely to be elicited by salient and thus glimpsed events, permitting a new classification decision to be made quickly. Our results also indicate that even a single glimpsed or masked ERP can be sufficient to produce an accurate (>70%) decoding decision (Fig. 3A–B); therefore, ERPC has the capacity to rapidly detect and amplify salient events that a listener might want to be amplified, allowing them to remain aware of new and relevant sources in the environment.

### 4.4. Distinction of glimpsed and masked events

In this study, we separately classified ERPs evoked by glimpsed and masked peakRate events because we wanted to distinguish between moments of the stimulus that evoked the most stereotyped responses. This proved effective, as increasing glimpse and mask thresholds improved the classifiers’ performances up to a point. However, it is not immediately clear that glimpsed and masked peakRate events should evoke distinct neural responses, as differences in glimpsed and masked speech processing were primarily shown for phonetic feature encoding (Raghavan et al., 2023). Nevertheless, another intracranial study indicated that phonetic features and peakRate events are represented in mostly overlapping sets of electrodes (Hamilton et al., 2021). Given their tight alignment with the onset of the syllabic nucleus (Oganian & Chang, 2019), peakRate events are thought to track syllable-level temporal structure. Thus, the glimpsed and masked peakRate events used here may represent a language-invariant method of identifying glimpsed and masked phonetic information at the syllabic level, which are expected to be distinctly encoded.

In addition to glimpsing, other speech measures that aid intelligibility have been proposed to segment speech for performing AAD, including the spectral transition measure (Tanaka et al., 2023) and the relative RMS intensity (Wang et al., 2021, 2022). In particular, RMS intensity and glimpsing are highly correlated, as more intense segments of speech are also more likely to be louder than the background; similarly, both high-RMS and glimpsed segments are each more likely to contain certain phonetic and syllabic features (Chen & Loizou, 2012; Cooke, 2006). Nevertheless, these two measures are distinct, as glimpsing accounts for dynamic overlap while RMS intensity is an estimate of the talker in isolation. More experiments will be needed to compare the relative efficacy of different forms of continuous speech segmentation on neural responses and AAD performance.

#### 4.4.1. Encoding of speech versus non-speech events

The possible linguistic basis of the encoding difference between glimpsed and masked speech raises the question of how non-speech sound sources might be detected by ERPC. On the one hand, the neural encoding of peakRate events and their amplitude were found to be similar for both speech and non-speech tones (Oganian & Chang, 2019). This indicates that the encoding, and thus decoding, of glimpsed peakRate events is likely similar for speech versus non-speech, as attention may work to modulate these stimuli. However, it remains unclear how masked peakRate events would be encoded for non-speech stimuli. An investigation of the phoneme restoration effect suggests that the recovery of masked speech results from the integration of sensory information with linguistic expectation and prediction (Leonard et al., 2016). Therefore, we may expect that non-speech sources that listeners can predict may be perceptually restored, as attention may work to enable the prediction of upcoming stimuli. However, it is unknown whether the perceptual restoration of speech and non-speech sources relies on a common neural substrate and, thus, whether these signals would be similarly decodable. Additional studies are needed to properly assess the influence of attention on the neural coding of non-speech signals in noise.

### 4.5. Use of invasive data and translation to non-invasive recordings

This study used iEEG data because intracranial high gamma envelopes have shown responses to peakRate events and distinct encoding of glimpsed and masked speech features. While most current methods for AAD are based on scalp EEG recordings, it is necessary to investigate iEEG for AAD. First, it is useful to establish an upper bound of performance. The upper bound set by iEEG will push forward overall maximum accuracies attained with AAD, helping researchers understand what is possible with such a system. Setting an upper bound of performance can also help disentangle the contributions of model capacity and data quality on AAD performance. Second, continuing advances in invasive neural recordings are leading to new possibilities with these methods. Developments in hardware have opened up the prospect of minimally-invasive surgeries for intracranial recordings (Oxley et al., 2016, 2021), and even smaller devices like the Neuropixel (Steinmetz et al., 2021), that can record reliably in speech cortices (Khanna et al., 2024; Leonard et al., 2023). Third, other speech BCIs use intracranial recordings (Luo et al., 2022; Metzger et al., 2022, 2023; Willett et al., 2023) even though recent work suggests speech neuroprostheses could be realized non-invasively (Défossez et al., 2023). Finally, the past decade of research into non-invasive methods for AAD has not proved fruitful enough to design a system capable of appropriately amplifying conversational speech. It remains unclear if non-invasive recordings will eventually prove sufficient or if invasive recordings will become sufficiently safe to be used more commonly. Regardless, both methods offer clear routes to the realization of a brain-controlled hearing aid and should continue to be investigated.

Further, it remains unclear if/how glimpsed and masked speech is distinctly encoded in non-invasively recorded LFPs. Nevertheless, direct-classification approaches to AAD denote a clear benefit to comparative approaches taken with CCA and other correlational methods. Therefore, it could be useful to extend classification approaches to non-invasive data. One option is to directly translate this method by characterizing spatiotemporal differences in the neural representation of glimpsed and masked speech events, computing average ERPs, and classifying them. However, the limited frequency range and low SNR of non-invasive recordings are likely to prevent this from being effective. A more promising option is to dynamically estimate TRFs for each talker in the scene using data from a single decoding window and classify the weights of the estimated TRFs to decode whether each talker is attended (Akram et al., 2017). This approach leverages the continuous-feature TRF estimation that is better suited for non-invasive recordings while allowing a classification approach that allows for different uses of attention and a reduced need to rapidly detect changes in attention. Further studies are needed to determine the efficacy of these approaches in non-invasive recordings.

### 4.6. Limitations

Beyond only being tested with invasive, as opposed to non-invasive, recordings, this method appears uniquely suited to iEEG recordings in STG. This is because STG contains attentionally modulated responses (Mesgarani & Chang, 2012), sites well-described by peakRate events (Oganian & Chang, 2019), and distinct responses to glimpsed and masked speech (Raghavan et al., 2023). Nevertheless, other parts of auditory cortex have displayed attentional selectivity, including anterolateral HG (J. O’Sullivan et al., 2019). However, because sites in anterolateral HG do not show responses to peakRate events (Hamilton et al., 2021), CCA may prove a more effective method for a subject with coverage in this region. Thus, this method is best suited for subjects with coverage over STG. The electrodes in this dataset were also predominantly located in the left hemisphere. This may have aided our decoding, as evidence indicates masked speech (Scott & McGettigan, 2013) and transitions from noisy to clean speech (Khalighinejad et al., 2019) are processed more in the left hemisphere. This electrode imbalance should not have affected decoding otherwise, as the loudspeaker was located directly in front of the participants to minimize any spatial effects on neural responses (Patel et al., 2022).

Further, the stimuli used in this study simply contained two talkers with no noise. It is unclear how the neural encoding of glimpsed and masked speech changes with more or different talkers or with noise. Therefore, it is unclear how differences in the acoustic environment might influence the hyperparameters of this decoding model, including the peakRate magnitude threshold, the glimpse SNR, and the glimpse and mask ratio thresholds. Additionally, the stimuli used here did not contain any real attention switches. While change in the orientation of attention is a cognitive process resulting in additional neural signatures beyond what would be seen by joining two segments of sustained attention, those signatures are not likely to be seen in sensory regions (Corbetta et al., 2008; Haro et al., 2022; Posner, 1980). Additionally, the transitions in CCA correlation shown here closely resemble the decoding of directed and at-will attention switching in EEG (Haro et al., 2022), suggesting that the simulated attention switching used here is a reasonable substitute for natural attention switching.

## 5. CONCLUSIONS

In conclusion, we present a new method for AAD based on the classification of ERPs to events in continuous speech. We show that the decoding of attention from these ERPs is enhanced by distinguishing between glimpsed and masked events, and this method of decoding outperforms CCA using the speech envelope. When simulating dynamic switches of attention, we find that ERPC detects switches of attention faster and results in more stable states of attention decoding. Finally, we show examples of possible instances of inattention or divided attention that ERPC could be capable of decoding, which the comparative method resulting from CCA correlations would be unable to decode. This work opens up a new direction in direct classification methods for AAD, allowing the decoding of different types of attention and changing the way attention switches are treated in an AAD system. Future work should investigate distinctions in glimpsed and masked speech encoding in non-invasively recorded LFPs and other types of classification methods for these data.

## Data Availability

The data that support the findings of this study are available upon request from N.M. The code for extracting the high gamma band envelope is available at http://naplab.ee.columbia.edu/naplib.html (Khalighinejad et al., 2017). The code for extracting the auditory spectrogram and peakRate events is available at https://naplib-python.readthedocs.io (Mischler et al., 2023).
